# The association between CD14 (C-159T) single-nucleotide polymorphism and Behcet’s syndrome and its clinical manifestations in Egyptian patients, an observational case–control genetic association study

**DOI:** 10.1007/s10067-025-07735-y

**Published:** 2025-11-04

**Authors:** Moustafa Ali Saad, Hala Ibrahem El Gendy, Olfat Shaker, Mariana Victor Philips, Yumn A. Elsabagh

**Affiliations:** 1 Rheumatology and Clinical Immunology Unit, Internal Medicine Department, Kasr Alainy Faculty of Medicine, Cairo University, Cairo, Egypt; 2 Medical Biochemistry and Molecular Biology Department, Kasr Alainy Faculty of Medicine, Cairo University, Cairo, Egypt; 3 Department of Internal Medicine, Faculty of Medicine, Cairo University, Cairo, Egypt

**Keywords:** Behcet syndrome, *C-159T* SNP, *CD14*, SNP

## Abstract

**Background:**

Cluster of differentiation 14 (CD14) molecules are immune cell surface molecules that bind and display lipopolysaccharides (LPSs) of gram-negative bacteria to Toll-like receptor 4 (TLR4), facilitating LPS-induced immune cell activation. The *CD14* promoter polymorphism *C-159T* is positively correlated with CD14, and homozygous carriers of the T allele have a significant increase in soluble and membrane-bound CD14.

**Objective:**

To assess the *C-159T* polymorphism in Behcet patients compared to controls, and to study its relationship with disease manifestations and activity.

**Methods:**

Fifty-one adult Egyptian patients with Behcet’s syndrome and another 51 healthy controls were recruited. Behcet’s syndrome activity was assessed. A blood sample was obtained from each participant for DNA extraction. The extracted DNA was used to determine the *C-159T* polymorphism in the *CD14* promoter gene *(rs2569190)* using real-time polymerase chain reaction.

**Results:**

The prevalence of the TT genotype was higher in Behcet patients (23.7%) in comparison to the controls (8%) (OR = 5.3, *P* value = 0.01). The prevalence of the T allele was higher in Behcet patients (49.1%) in comparison to the controls (31.4%) (OR = 2.1, *P* value = 0.01). The skin erythema was found to be significantly (*P* value = 0.003) higher in frequency among the TT genotype (58.3%) compared to the CT genotype (26.9%). Moreover, the skin pustules were found to be significantly (*P* value = 0.01) higher in frequency among the TT genotype (41.6%) compared to the CT genotype (11.5%).

**Conclusion:**

*CD14 (C-159T)* polymorphism is associated with an increased risk of developing Behcet’s syndrome and is correlated with its dermatological manifestations.

**Table:** 

**Key points** • *Behcet’s syndrome is a variable-vessel vasculitis in which aberrant innate immunity is integral to the pathogenesis of the disease*. • *CD14 molecules recognize pathogens with subsequent activation of innate immunity*. • *The CD14 promoter gene C-159T single-nucleotide polymorphism increases the susceptibility to Behcet’s syndrome*. • *The C-159T polymorphism correlates with skin manifestations of Behcet’s syndrome*.

## Introduction

Behcet syndrome (BS) is a variable-vessel vasculitis that is characterized by orogenital ulcerations, pathergy, and eye disease [1].

One of the early proposed theories is the role of innate immunity in the pathogenesis of BS [2]. Toll-like receptors (TLRs) can recognize a wide spectrum of pathogen-associated molecular patterns (PAMPs), with subsequent production of a wide range of immune stimulatory cytokines. Abnormal activation of TLRs may result in a relentless inflammatory response, which is central to many autoimmune diseases. Toll-like receptor 4 (TLR4) is capable of recognizing lipopolysaccharides (LPSs) of gram-negative bacteria [3].

The cluster of differentiation 14 (CD14) molecules are cell surface molecules that are co-receptors expressed over dendritic cells, monocytes, macrophages, and granulocytes, and their function is to bind and display LPSs of gram-negative bacteria to TLR4, facilitating LPS-induced macrophage, monocyte, and neutrophil activation [4].

The *CD14* promoter polymorphism *C-159T* is a recognized single-nucleotide polymorphism (SNP) present in the proximal *CD14* promoter at position − 159. It was found that homozygous carriers of the T allele have a significant increase in soluble and membrane-bound CD14 [5].

The *C-159T* SNP is associated with diseases like atopic asthma [6] and allergic rhinitis [7], while it is not associated with familial Mediterranean fever, amyloidosis [8], or sepsis [9]. It has recently been studied in systemic lupus erythematosus (SLE) and has been proven to be associated with an increased predisposition to the development of SLE and lupus nephritis, highlighting the possible role of innate immunity in SLE [10].

The role of CD14, as a part of the innate immune response, in BS was studied as early as 1996, and soluble CD14 was found to be raised in BS patients, compared with controls, suggesting a possible infectious trigger of BS. The findings of this study indicated the presence of hyperactive monocytes and macrophages in Behcet’s patients. At this time, the cause of monocyte or neutrophil activation in BS was still unknown [11]. Although a previous study showed that there was no association between *CD14* SNPs at the − 159 position and BS [12], this association was not studied in the Egyptian population before.

We aimed to study the association between the *C-159T* SNP and BS in Egyptian patients, in comparison to the general population, and to correlate the *C-159T* SNP with various manifestations and activity of the disease.

## Methods

### Population of the study

Fifty-one adult Egyptian patients were recruited from Cairo University hospitals and the outpatient Behcet’s clinic in the period from May 2021 to September 2021. The recruited individuals were diagnosed with Behcet’s Syndrome according to the International Study Group Criteria for Behçet Disease (ISG) (1990) [13], with another 51 age- and sex-matched healthy controls. This study was approved by the research ethics committee of Kasr Alainy Faculty of Medicine, Cairo University, Cairo, Egypt, on 25/4/2021 (Code: MS-41–2021). All of the participating subjects gave their written informed consent to participate.

**Fig. 1:**
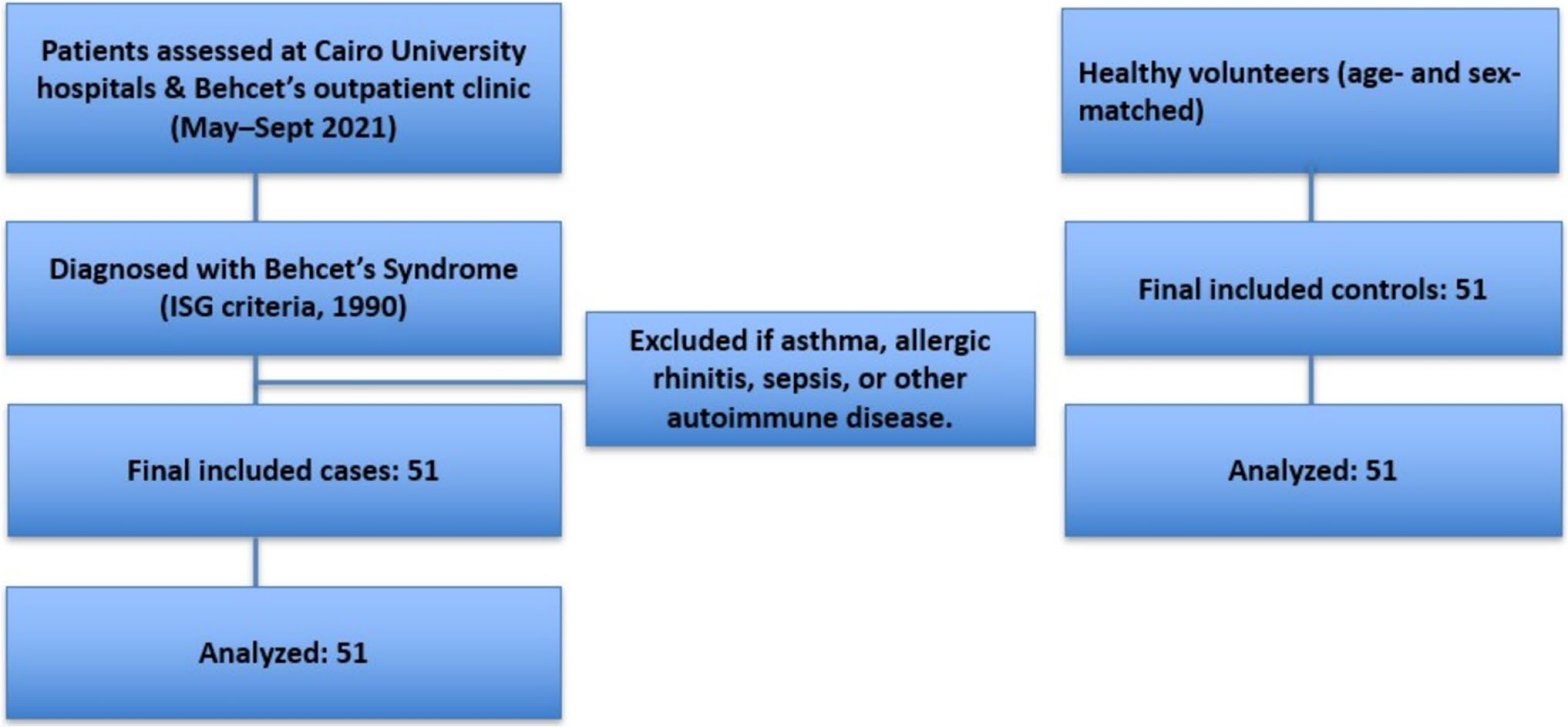
Flow diagram showing the selection process of patients and controls. ISG, International Study Group

Patients diagnosed with atopic asthma and allergic rhinitis, patients in active sepsis, and patients previously diagnosed with any other autoimmune disease were excluded from the study. The selection process is illustrated as a flow diagram in Fig. 1.

The sample size was estimated prior to the start of the study using GPower software version 3.1.9.7 (http://www.gpower.hhu.de/en.html), informed by data from preceding studies. The calculation indicated that a total of 102 participants (51 cases and 51 controls) were required, based on a 5% margin of error and an 80% confidence level.

The activity of Behcet’s syndrome was assessed in the 51 cases, using two activity indices: Behcet’s Disease Current Activity Form (BDCAF) [14] and the Behcet Syndrome Activity Score (BSAS) [15].

### Genotyping

Three milliliters of blood was obtained from each participant in an EDTA tube for DNA extraction. DNA was extracted from whole blood using the QIAamp kit supplied by Qiagen (Qiagen, Hilden, Germany, catalogue number 51306) according to the manufacturer’s specifications. DNA was quantified by Nanodrop 2000 and stored at − 20 °C for PCR amplification.

A reaction mix was prepared as follows: 12.5 μL of Taq genotyping master mix supplied by Qiagen (Qiagen, Hilden, Germany, catalogue number 201443), 1.25 μL of the assay *rs2569190* (PN4351379), and 20 ng DNA in a total volume of 25 μL. The cycling program was as follows: one cycle for 10 min at 95 °C followed by 40 cycles of 95 °C for 30 s and 60 °C for 1 min using Rotor gene (Qiagen, Hilden, Germany).

### Statistical analysis

Data was analyzed using Microsoft Excel XP and Graphpad Prism version 5.0 software (USA). The normally distributed data was expressed as mean ± SEM, whereas variables with a skewed distribution were presented as median (interquartile range). The statistical comparisons between different groups were carried out using unpaired Student’s *t*-test for normally distributed data and Mann–Whitney *U* test and Kruskal–Wallis test for non-normally distributed data. Categorical data was represented by frequency and percentage, as well as compared by chi-square (*X*^2^) test and odds ratio. The correlation between variables was evaluated using Spearman’s rank or Pearson’s correlation coefficient test (2–2-tailed). The level of significance was identified at *P* < 0.05.

## Results

**Fig. 2:**
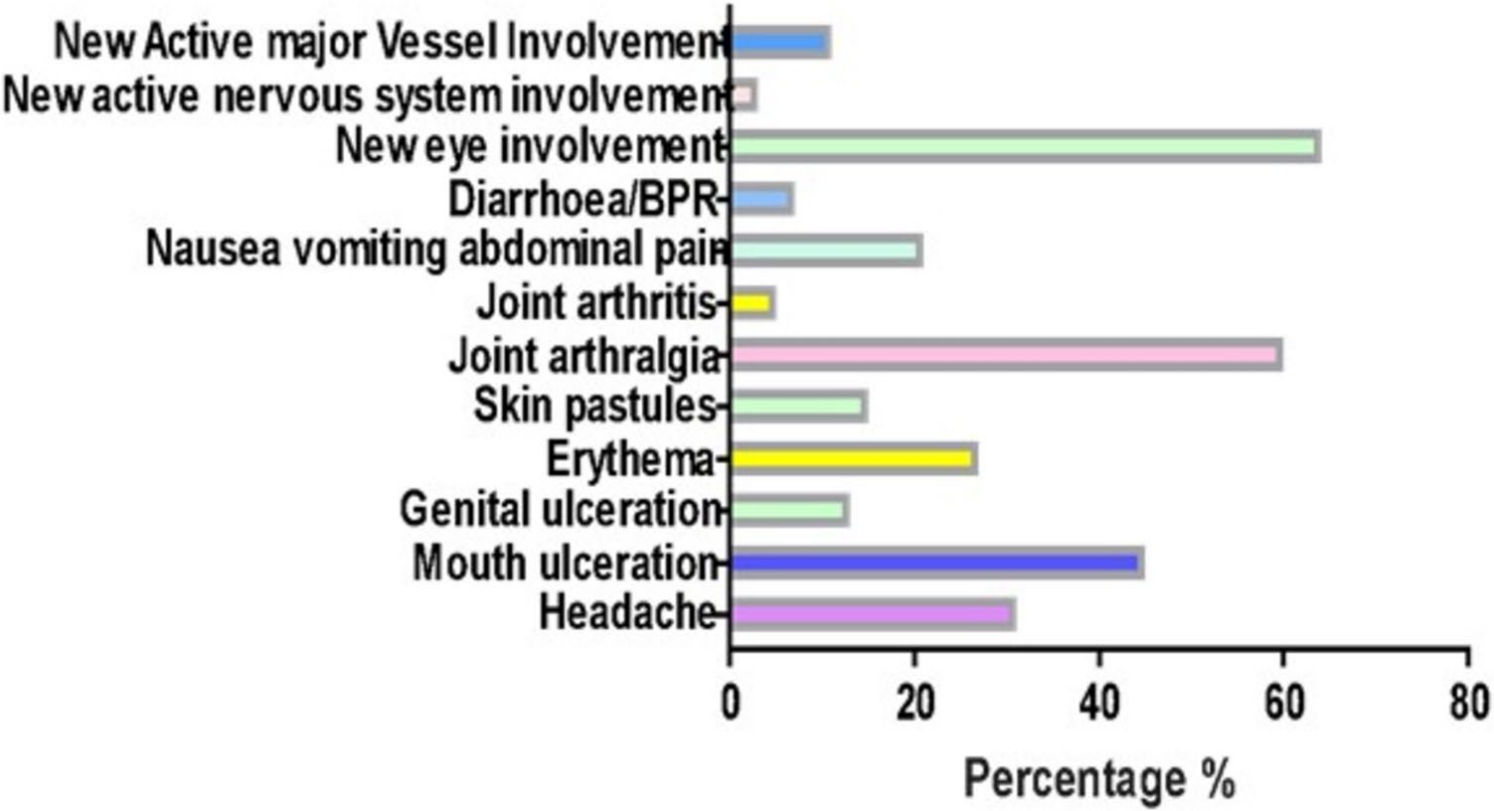
Features of Behcet patients

Fifty-one Behcet patients and 51 healthy controls were enrolled in this study. The mean age of Behcet patients (*n* = 51) was 33.1 ± 8.13 years, while the control group (*n* = 51) had a mean age of 35.2 ± 6.04 years (*P* value = 0.14). In terms of gender, there were 8 females and 43 males in the Behcet group, and 14 females and 37 males in the control group (*P* value = 0.149). The most frequent manifestation of disease activity during the last month before recruitment was new eye involvement (64.7%), followed by arthralgia (60.8%) and mouth ulcerations (45.1%) (Fig. 2).

Regarding the activity of the disease, the median activity scores were 6 out of 10 on the Patient General Assessment (PtGA), 5 out of 10 on the Physician General Assessment (PGA), 7 out of 20 on the BDCAF, and 25 out of 100 on the BSAS.

**Fig. 3:**
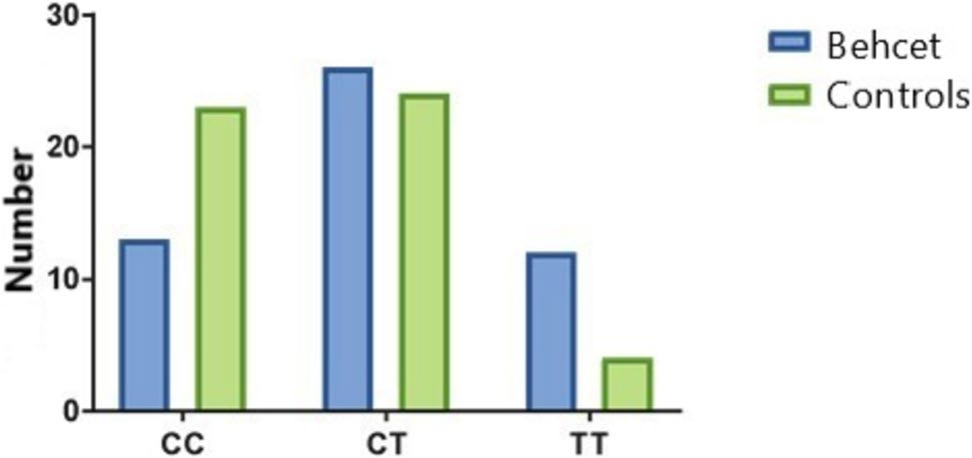
Prevalence of different genotypes among the studied groups. Data were expressed as numbers

**Fig. 4:**
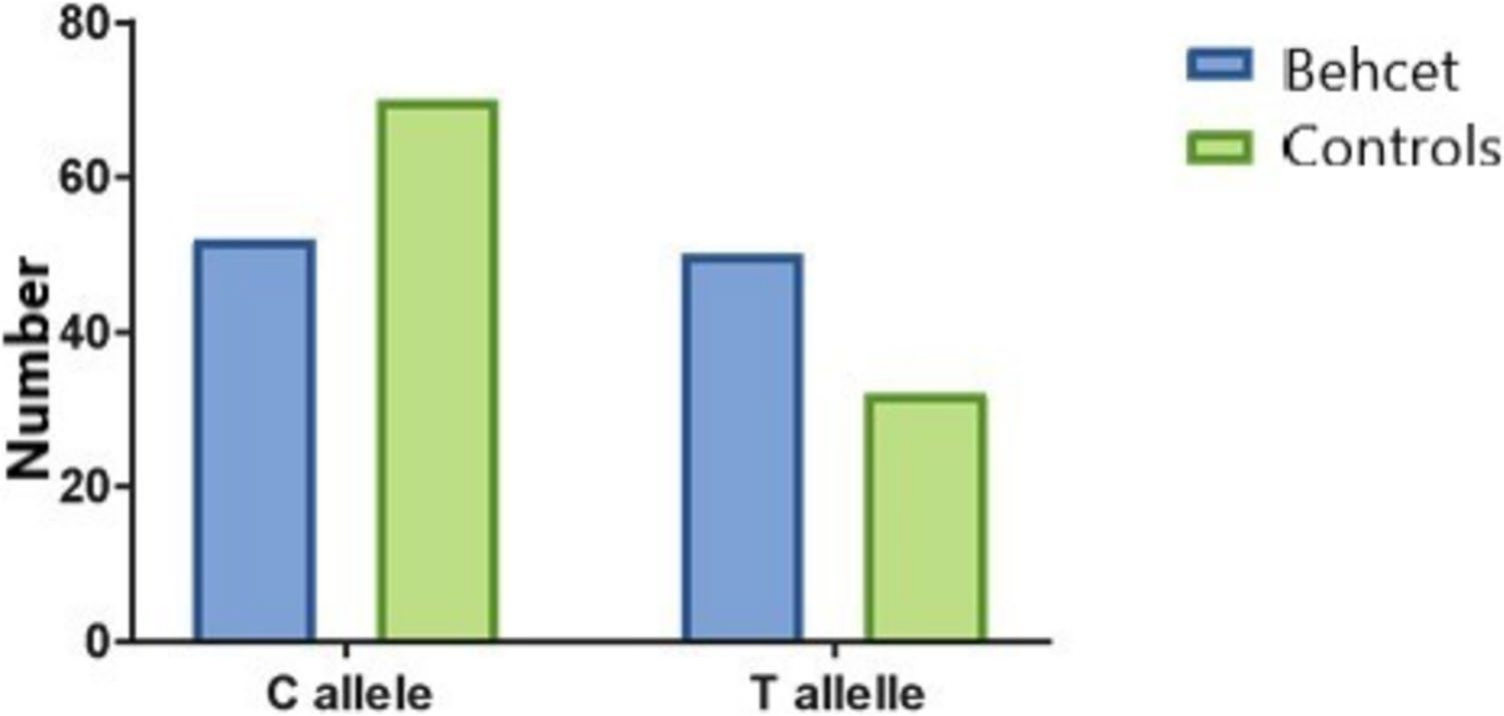
Prevalence of different alleles among the studied groups. Data were expressed as numbers

The prevalence of different genotypes and alleles among the studied groups is shown in Table 1 (Figs. 3 and 4). The prevalence of the TT genotype was higher in Behcet patients (23.7%) in comparison to the controls (8%) (OR = 5.3, *P* value = 0.01). The prevalence of the T allele was higher in Behcet patients (49.1%) in comparison to the controls (31.4%) (OR = 2.1, *P* value = 0.01).

**Table 1:** Prevalence of different genotypes and alleles among the studied groups

Genotypes	Behcet patients (*n* = 51)	Control subjects (*n* = 51)	Odds ratio	95% CI	*P* value
*CC*	13 (25.4%)	23 (45%)	Ref	1	
*CT*	26 (50.9%)	24 (47%)	1.9	0.7–4.6	0.14
*TT*	12 (23.7%)	4 (8%)	5.3	1.4–19.8	**0.01**
*C allele*	52 (50.9%)	70 (68.6%)	Ref	1	
*T allele*	50 (49.1%)	32 (31.4%)	2.1	1.1–3.7	**0.01**

Regarding the association of *CD14 (C-159T)* polymorphism with the clinical manifestations of BS, of all the disease manifestations, the skin erythema was found to be significantly (*P* value = 0.003) higher in frequency among the TT genotype (58.3%) compared to the CT genotype (26.9%), while not present among the CC genotype group at all. Also, the skin pustules were found to be significantly (*P* value = 0.01) higher in frequency among the TT genotype (41.6%) compared to the CT genotype (11.5%), while not present among the CC genotype group at all. On the other hand, no significant association could be found between any other manifestation of activity, including eye, neurological, and vascular involvement, and the *C-159T SNP* (Table 2).

**Table 2:** Features of Bechet patients among different genotypes

Features	*CC *(*n* = 13)	*CT *(*n* = 26)	*TT *(*n* = 12)	*X*^*2*^	*P*
Headache	3 (23%)	10 (38.4%)	3 (25%)	1.15	0.57
Mouth ulceration	8 (61.5%)	11 (42.3%)	4 (33.3%)	2.17	0.35
Genital ulceration	3 (23%)	3 (11.5%)	1 (8.3%)	1.34	0.56
Erythema	0 (0%)	7 (26.9%)	7 (58.3%)	10.8	**0.003 **
Skin pustules	0 (0%)	3 (11.5%)	5 (41.6%)	7.4	**0.01**
Arthralgia	6 (46.1%)	16 (61.5%)	9 (75%)	2.1	0.33
Arthritis	1 (7%)	1 (3.8%)	1 (8.3%)	1	0.79
Nausea, vomiting, abdominal pain	2 (15.3%)	6 (23%)	3 (25%)	0.48	0.82
Diarrhea, bleeding per rectum	2 (15.3%)	1 (3.8%)	1 (8.3%)	1.86	0.43
New eye involvement	9 (69.2%)	18 (69.2%)	6 (50%)	1.49	0.47
New active nervous system involvement	1 (7%)	1 (3.8%)	0 (0%)	1.16	1
New active major vessel involvement	1(7%)	4 (15.3%)	1 (8.3%)	0.56	0.86

No significant association could be found between disease activity and the studied *C-159T SNP* (Table 3).

**Table 3:** Activity scores of Behcet patients among different genotypes

*Scores*	*CC* *(n* = *13)*	*CT* *(n* = *26)*	*TT* *(n* = *12)*	*P* value
Patient Global Assessment (PtGA)	6.5 (3.5–9.75)	8 (5–10)	5 (2.5–9.5)	0.7
Physician Global Assessment (PGA)	5 (4 −9)	5 (4.75–9)	5 (2.5–6.75)	0.82
BDCAF (Transformed index score on interval scale 0;20)	5 (4–7)	7 (5–8)	7 (5–9)	0.54
BSAS score/100	25 (17–39)	29.5 (19.75–42)	17.5 (11.2–33.5)	0.19

Data are expressed as median (25th–75th percentiles). Data were calculated using the Kruskal–Wallis test

*BDCAF* Behcet’s Disease Current Activity Form, *BSAS* Behcet Syndrome Activity Score

The severity of Behcet activity was categorized into the following: mild group (BDCAF 0–5, BSAS 0–25), moderate group (BDCAF 6–10, BSAS 26–50), severe group (BDCAF 11–15, BSAS 51–75), and very severe group (BDCAF 16–20, BSAS 76–100), but no significant association could be found between those groups and the studied SNP.

## Discussion

Our study proved a significant correlation between the TT genotype, the CT genotype, and the T allele of *CD14 (C-159T)* polymorphism and Behcet syndrome. It also proved a significant correlation between *C-159T* SNP and skin manifestations of the disease. This possibly highlights the role of the innate immune system in the pathogenesis of BS.

Neutrophil hyperreactivity to LPSs of gram-negative bacteria and neutrophil hyperchemotaxis were considered as possible pathological elements in BS in a previous study [16]. This study found that sCD14 levels were correlated with BS occurrence, which was consistent with our findings. On the other hand, sCD14 levels were found to be correlated with BS activity as assessed by BDCAF score, a finding that is apparently contradictory to our result of no association between CD14 *C-159T* polymorphism and disease activity. This discrepancy may not only be attributed to ethnic differences, but may also point to different genetic polymorphisms affecting CD14 levels and yet to be discovered.

One possible link between the CD14 pathway and BS is the nuclear factor kappa B (NFKB) pathway. CD14 activation leads to the activation of multiple intracellular pathways, of which NFKB activation is important in activating the innate immune response [17]. The NFKB pathway was previously studied in BS and found to be involved [18]. This may occur through different mechanisms, including long noncoding RNA maternally expressed gene 3 (MEG3), long noncoding RNA musculoaponeurotic fibrosarcoma oncogene family, protein G antisense 1 (MAFG-AS1), and microRNA 147-b axis [19] and microRNA-9 [20].

A higher prevalence of the *C-159T* SNP has been observed in systemic lupus erythematosus (SLE) and has been proven to be associated with increased predisposition to the development of SLE and lupus nephritis, highlighting a possible role of innate immunity in systemic lupus. The functional relevance of this finding was also demonstrated in SLE through assessment of sCD14 levels that were found to be high in patients with SLE and lupus nephritis [10]. In our study, we proved the same finding regarding the *C-159T* SNP in BS, but we did not investigate the functional relevance of this SNP through measuring sCD14 levels, a point of research that still needs investigation. However, as mentioned earlier, we already have early reports assessing CD14 in BS [16] that are considered complementary to our study.

The significant correlation between *C-159T* SNP and skin manifestations of BS is consistent with the previously described theory of neutrophil hyperfunction as a pivotal part of leukocytoclastic vasculitis occurring in papulopustular eruptions of BS [21, 22].

It was found that the main pathogenic factors in the dermatological lesions of BS were leukocytoclastic vasculitis and perivascular neutrophilic infiltration. Those findings demonstrated the role of the innate immune response in cutaneous lesions of BS [21].

Another study by Alpsoy et al. [23] supports our results too. This study showed that a basic feature of papulopustular lesions (PPLs) of patients with BS is leukocytoclastic vasculitis. Lesional specimens from PPLs showed infiltration of predominantly leukocytes around the vessels.

Another Turkish study [24] concluded that the pustular lesions of BS were not sterile, with unusual bacteria in unusual sites. Whether these pustules were secondarily infected or the infections played a pathogenic role in the development of pustular lesions remains to be determined. Moreover, another study showed that the pathergy reaction is minimized via surgical cleaning before introducing the needle into the skin [25]. Calgüneri and colleagues demonstrated decreased pustular lesions in BS patients through the use of prophylactic antibiotics [26]. Our results can be more understandable in view of this study.

In another study, the CD14 (− 159)*T allele seemed to be significantly associated with susceptibility to SLE and arthritis occurrence in SLE patients but not in RA patients [27]. However, in our study, we found no significant association between the arthritis activity of BS and the mutant allele of the *C-159T* SNP, possibly demonstrating different pathogenesis of joint involvement between BS and other rheumatologic conditions.

On the other hand, another two different studies showed no association between this SNP and RA occurrence or severity [28, 29].

Although previous Genome-Wide Association Studies (GWAS) detected no relation between CD14 SNPs and BS [30], our study is a preliminary one performed on a new ethnic group that was not studied in any previous GWAS.

Regarding the background data, most of our cases were males, representing the predominance of the disease in males versus females. The most frequent manifestations of disease activity among our patients were new eye involvement (64.7%), followed by arthralgia (60.8%), then mouth ulcerations (45.1%). In a previous Egyptian study, throughout the study period, the commonest manifestations were oral ulcers (100%), followed by genital ulcers (96.8%), vascular lesions (57.1%), cutaneous lesions (55.5%), ocular lesions (47.6%), and joint involvement (36.5%) [31]. According to another Egyptian study, all patients had oral ulcers, while genital ulcers were present in 80%. The eyes were involved in 78.9%, and cutaneous manifestations were present in 59.8%, arthritis in 34.7%, neuropsychiatric manifestations in 13.9%, gastrointestinal tract involvement in 11.5%, and deep venous thrombosis in 23.1% [32]. The differences are mostly because our study calculated the frequencies of disease activity manifestations in the preceding month, not throughout the whole course of the disease.

Our results contradict the results of a previous study that did not find any statistically significant association between CD14 SNPs at the − 159 position and BS [12]. However, this previous study was done in different countries, probably highlighting different genetic backgrounds in different populations.

Although our study concluded an association between the *CD14* promoter gene *C-159T* SNP and BS occurrence, these findings should be interpreted with caution given the relatively small sample size, and further studies with larger cohorts are required to validate our results. Moreover, there is a need to study the functional relevance of this SNP by measuring the sCD14 level in the serum and relating it to BS activity and its various clinical manifestations.

In addition, using BDCAF and BSAS activity scores, our study assessed the activity of the disease in the preceding month before the date of assessment, but not the frequencies of cumulative disease manifestations or disease damage. If those were assessed, other associations might have appeared.

To conclude, the present study demonstrated preliminary evidence for an association between BS and the TT genotype, the CT genotype, and the T allele of *CD14 (C-159T)* polymorphism, but did not find any significant association between the studied SNP and global disease activity. Of all disease manifestations, the skin erythema and pustules were found to be more frequent among the TT genotype (58.3% and 41.6% respectively) compared to the CT genotype (26.9% and 11.5% respectively), while not present among the CC genotype group at all. In contrast, no significant association could be found between any other manifestation of activity and the *C-159T* SNP.

## Data Availability

The authors confirm that the data supporting the findings of this study are available within the article; any extra data needed are available from the corresponding author upon reasonable request.

## Abbreviations

*BDCAF*: Behcet’s Disease Current Activity Form
*BS*: Behçet’s syndrome
*BSAS*: Behcet Syndrome Activity Score
*CD14*: Cluster of differentiation 14 molecules
*GWAS*: Genome-Wide Association Studies
*ISG*: The International Study Group Criteria for Behçet Disease
*LPSs*: Lipopolysaccharides
*MAFG-AS1*: Musculoaponeurotic fibrosarcoma oncogene family, protein G antisense 1
*MEG3*: Maternally expressed gene 3
*NFKB*: Nuclear factor kappa B
*PAMPs*: Pathogen-associated molecular patterns
*PGA*: The Physician General Assessment
*PPLs*: Papulopustular lesions
*PtGA*: The Patient General Assessment
*SLE*: Systemic lupus erythematosus
*SNP*: Single nucleotide polymorphism
*TLR4*: Toll-like receptor 4
*TLRs*: Toll-like receptors
